# Supplementary material for: Verkko2 integrates proximity-ligation data with long-read De Bruijn graphs for efficient telomere-to-telomere genome assembly, phasing, and scaffolding
Source: Genome Res. 2025 Jul;35(7):1583–94. doi: 10.1101/gr.280383.124 (PMC12212074; doi:10.1101/gr.280383.124)
Supplement: Supplement 2 [file Supplemental_Materials.pdf]

SUPPLEMENTARY MATERIALS FOR: VERKKO2: INTEGRATING PROXIMITY LIGATION DATA WITH LONG-READ DE BRUIJN GRAPHS FOR EFFICIENT TELOMERE-TO-TELOMERE GENOME ASSEMBLY, PHASING, AND SCAFFOLDING

TABLE OF CONTENTS

Table S1
Table S2
Table S3
Table S4
Figure S1
Figure S2
Figure S3
Supplementary methods S1

1. TABLES

	sheep	chicken	HG002	HG00733
Genome size (Gb)	2.7	1.1	3.1	3.1
Het Rate (%)	0.988	0.950	0.262	0.114
number of chrs (2n)	54	78	46	46
HiFi				
N50	23,393	21,912	13,607	15,537
Total Bases (Gb)	213.33	109.35	198.30	183.05
ONT				
Bases in reads >=100 kb (Gb)	105.09	17.75	95.60	170.84
Total Bases (Gb)	497.32	177.00	247.19	271.14
Hi-C				
Total Bases (Gb)	65.19	116.46	125.87	198.95

Table S1. Information about datasets used for benchmarking. Heterozygosity level was estimated with genomescope [1] using the HiFi reads, except for chicken, where genomescope crashed on HiFi data. For that sample Hi-C Illumina reads were used for estimation. Heterozygosity of the heterogametic samples (sheep, chicken, HG002) can be overestimated with this tool.

Species	T2T scf	T2T ctgs	Hamming error	Switch error	QV	Missing genes	Missing genes (no sex chr)	CPU Hours	Peak Memory
**Sheep**									
Verkko2 Hi-C	**31**	**24**	**0.85**%	**0.58**%	54.17	1.37%	**0.06%**	3725.80	206
Verkko2 trio	23	20	**0.85**%	0.95%	54.17	**1.36**%	**0.06%**	**2897.02**	203
Hifiasm Hi-C	17	16	0.86%	0.95%	57.25	1.37%	**0.06%**	4342.36	381
Hifiasm trio	20	19	**0.85%**	0.94%	**57.46**	**1.36**%	**0.06%**	4046.67	396
Verkko1 trio	20	15	**0.85**%	0.95%	55.85	**1.36%**	**0.06%**	7181.07	**196**
**Chicken**									
Verkko2 Hi-C	34	21	0.58%	**0.13%**	45.13	3.12%	1.07%	870.31	84
Verkko2 trio	32	25	0.58%	**0.13%**	**45.17**	2.52%	0.47%	**673.58**	85
Hifiasm Hi-C	35	32	2.01%	0.33%	40.34	**2.20%**	**0.15%**	1252.55	202
Hifiasm trio	**36**	35	**0.41%**	0.34%	40.25	2.25%	0.20%	1268.15	203
Verkko1 trio	25	23	0.43%	0.30%	39.88	2.56%	0.49%	2150.19	**69**
**HG002**									
Verkko2 Hi-C	**40**	21	0.39%	**0.41%**	53.87	1.61%	**0.09%**	1736.25	**164**
Verkko2 trio	32	**22**	**0.38%**	**0.41%**	53.89	**1.60%**	**0.09%**	**1394.57**	**164**
Hifiasm Hi-C	17	9	0.51%	0.47%	55.12	1.64%	0.13%	2347.53	325
Hifiasm trio	18	10	0.46%	0.48%	**55.29**	1.61%	0.11%	2330.15	328
Verkko1 trio	21	8	0.46%	0.50%	51.52	2.64%	1.13%	9794.19	165
**HG00733**									
Verkko2 Hi-C	**41**	**26**	0.75%	**0.79%**	53.86	**0.09%**	**0.09%**	2112.23	165
Verkko2 trio	33	23	**0.74%**	**0.79%**	53.82	**0.09%**	**0.09%**	**1518.56**	165
Hifiasm Hi-C	22	14	2.73%	0.86%	**56.63**	0.10%	**0.09%**	2552.40	283
Hifiasm trio	23	15	0.81%	0.87%	56.52	0.10%	0.10%	2629.20	275
Verkko1 trio	19	11	0.78%	0.83%	51.97	0.62%	0.61%	8345.69	**162**

Table S2. Comparison of tested assemblers on human and non-human data on all metrics. Scaffolds $<$ 100 kb were discarded for all metrics. T2T scaffolds are scaffolds longer than 5 Mb that contain telomeres (detected by seqtk telo) on both ends. Hamming error rate, switch error rate, and QV were calculated with yak. Missing genes count were calculated with compleasm v0.2.6 (haplotypes evaluated independently, average values reported). All assemblers were run on the NIH Biowulf cluster. Best values for each metrics and sample are highlighted in bold. Verkko2 Hi-C has the highest T2T scaffold count with the exception of chicken where it is two less than the best. Verkko2 trio has the lowest runtime across all datasets, followed by Verkko2 Hi-C. While Verkko1 has the lowest memory usage, Verkko2 only modestly increases memory while reducing runtime $2.5 - 7$-fold.

	Verkko2 Hi-C	Verkko2 trio	Verkko1 trio	Hifiasm Hi-C	Hifiasm trio
NA50	133.576	**133.990**	130.098	95.008	101.268
NA90	45.180	**45.332**	36.924	39.271	39.310
misassemblies	97	**63**	119	277	163
Genome fraction %	**99.91**	**99.91**	99.7	98.60	99.78
local misassemblies	216	**214**	550	816	420
mismatches per 100Kbp	0.43	**0.41**	1.33	2.96	1.06
indels per 100Kbp	0.77	**0.75**	0.92	1.28	0.87
N's per 100 kbp	34.15	**18.21**	55.43	185.77	81.77

Table S3. QUAST accuracy evaluation of all tested assemblies on HG002 dataset, using HG002 genome release v1.1 as a reference. Scaffolds $<$ 100 kb were discarded for all metrics. NA50 and NA90 are reported in Mb. Best values among all assemblers is highlighted in bold.

Sample ID	T2T ctg	T2T scf	Hamming rate	Switch rate	QV	Missing	Missing (no sex chr)	Missing №	Dup №
**HG00621**	18	36	0.41%	0.38%	57.00	1.72%	0.17%	473	187
**HG00735**	10	30	0.65%	0.71%	53.26	0.21%	0.20%	57	205
**HG00741**	18	43	0.66%	0.65%	57.19	0.17%	0.17%	48	198
**HG01106**	19	40	0.48%	0.37%	52.29	1.70%	0.17%	469	186
**HG01175**	24	38	0.74%	0.62%	56.17	0.20%	0.20%	56	193
**HG01258**	22	37	0.36%	0.40%	56.06	1.70%	0.17%	468	189
**HG01891**	28	38	0.53%	0.52%	57.28	0.16%	0.16%	44	189
**HG01952**	28	39	0.49%	0.51%	57.24	1.72%	0.19%	473	193
**HG02148**	14	38	0.73%	0.77%	57.23	0.25%	0.25%	69	214
**HG02486**	27	42	0.33%	0.36%	54.94	1.69%	0.16%	465	183
**HG02559**	26	45	0.52%	0.53%	54.67	0.16%	0.16%	45	185
**HG02572**	27	42	0.32%	0.38%	56.80	1.69%	0.16%	466	185
**HG02622**	21	37	0.76%	0.65%	53.74	0.16%	0.16%	44	190
**HG02630**	22	41	0.53%	0.66%	51.56	0.16%	0.16%	45	193
**HG02886**	20	39	0.62%	0.60%	50.94	0.19%	0.19%	52	195
**HG03453**	21	39	1.48%	0.65%	51.29	0.16%	0.16%	45	196
**HG03540**	21	42	0.62%	0.80%	49.82	0.18%	0.18%	49	187
Verkko2 Hi-C Median	21	39	0.53%	0.60%	54.94	0.20%	0.17%	56	190
Hifiasm yr1 Median	0	0	0.71%	0.61%	53.57	0.24%	0.23%	67	216

Table S4. HPRC Yr1 assembly metrics for Verkko2 Hi-C. T2T scaffolds are scaffolds longer than 5 Mb that contain telomeres (detected by seqtk telo) on both ends. Hamming error rate, switch error rate, and QV were calculated with yak. Missing genes count were calculated with compleasm v0.2.6 (haplotypes evaluated independently, average values reported for percentages).

2. FIGURES

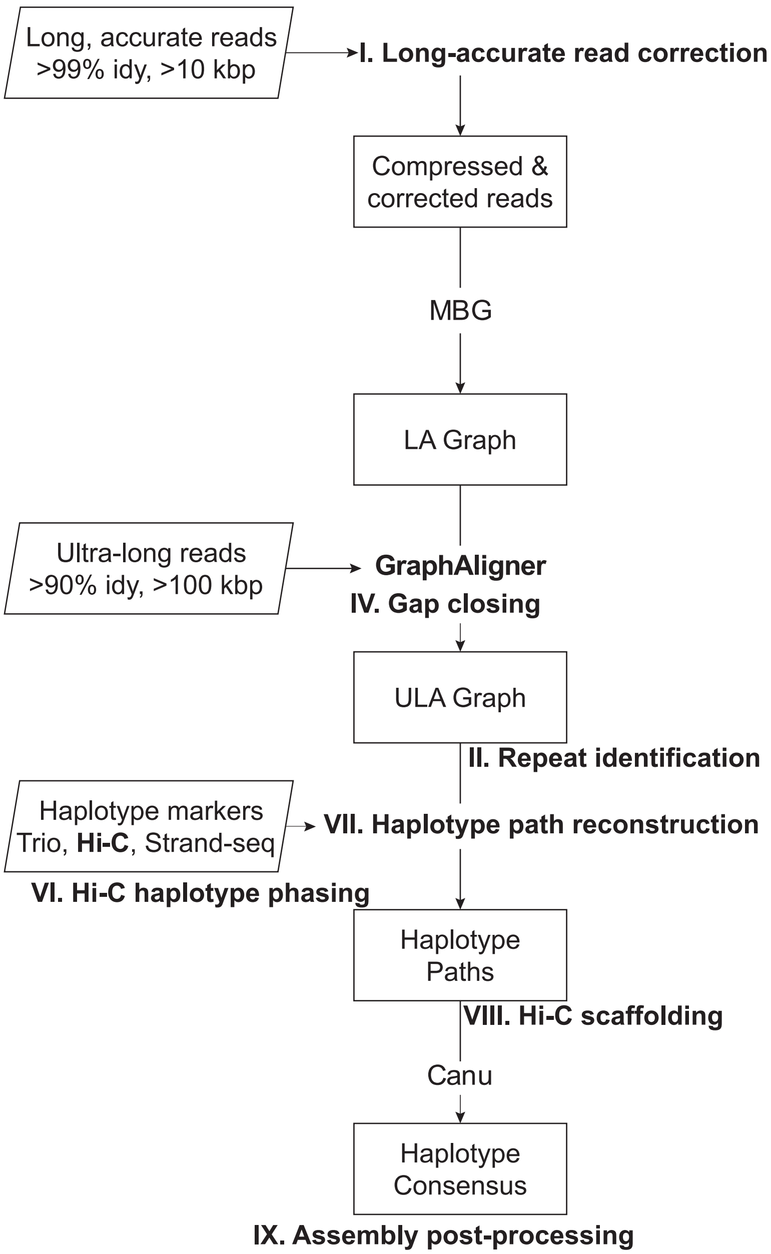

Fig. S1. Verkko1 pipeline graphical representation, adapted from [2]. Stages modified in Verkko2 are labeled with roman numerals and are described in the corresponding subsections of Methods: I: Long-accurate read correction, II+III: Repeat identification and better assembly for telomeres, IV: Gap closing, VI: Hi-C haplotype phasing, VII: Haplotype path reconstruction, VIII: Hi-C scaffolding, and IX: Assembly post-processing.

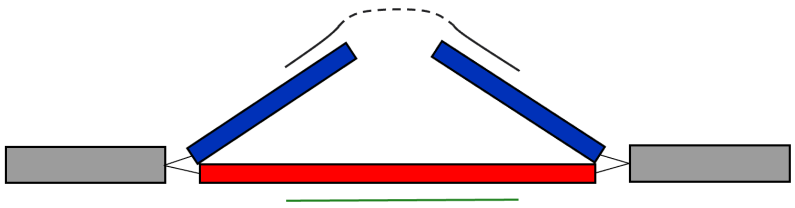

Fig. S2. An example of two possible alignments of an ONT read to a gapped region of the LA graph. The grapy nodes are homozygous and used by both haplotypes. The red and blue nodes correspond to the maternally-inherited and paternally-inherited haplotype, respectively. The paternally-inherited haplotype has a gap due to a coverage dropout in the LA data. The correct alignment is represented by two solid black lines connected by a dash. The middle part of the read sequences comes from a region absent in the LA graph and is represented by dashed black line. The alignment to the alternate haplotype is represented by solid green line. This haplotype does not have missing sequence in the LA graph. Although the alternate haplotype may have lower identity, it can have a higher score and be selected because it provides a single alignment with more bases covered by the alignment and no gap penalties.

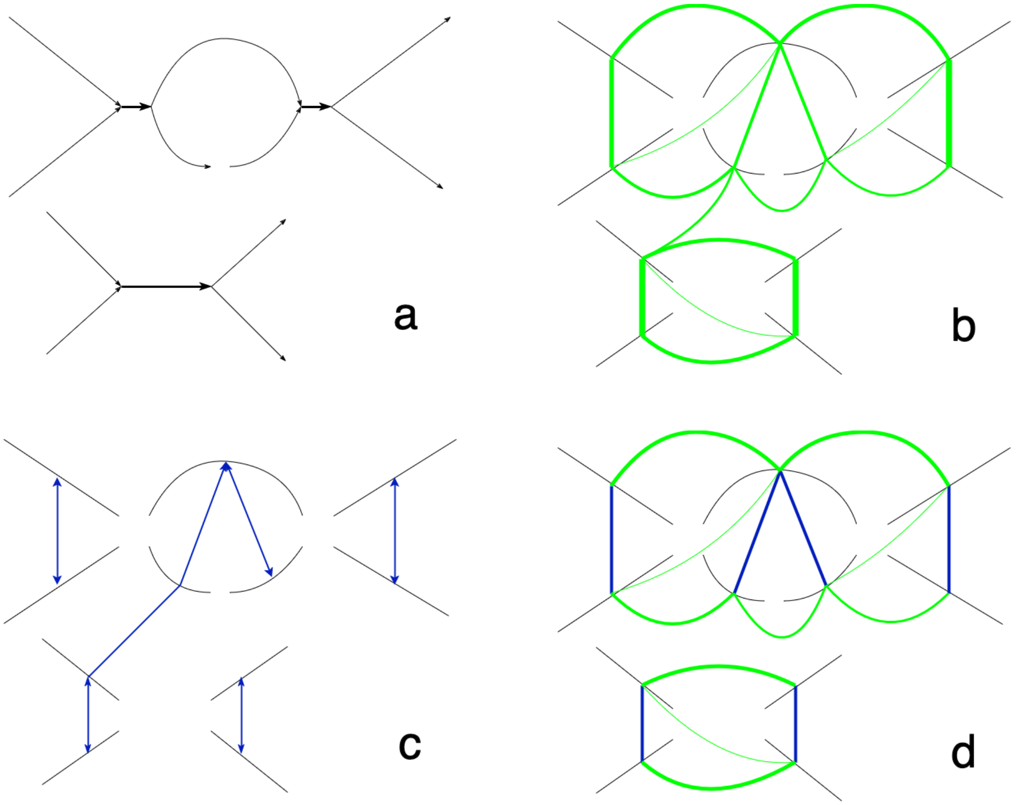

Fig. S3. Steps of Hi-C the phasing algorithm: a) Initial ULA assembly graph with two connected components. b) The Hi-C Graph prior to any filtering. The thickness of edges correspond to the number of Hi-C read pairs mapping to both nodes c) The MatchGraph, with alignment matches shown in blue. The arrow on the edges indicates best matches. For example an arrow pointing from node x to node y to show that y is the best match for x. d) The filtered Hi-C Graph. The MatchGraph edges are used to generate large negative weights (shown in blue). Non-best edges (e.g. connecting the two components) are set to have a 0 value and are dropped in this figure. Remaining Hi-C edges are shown in green.

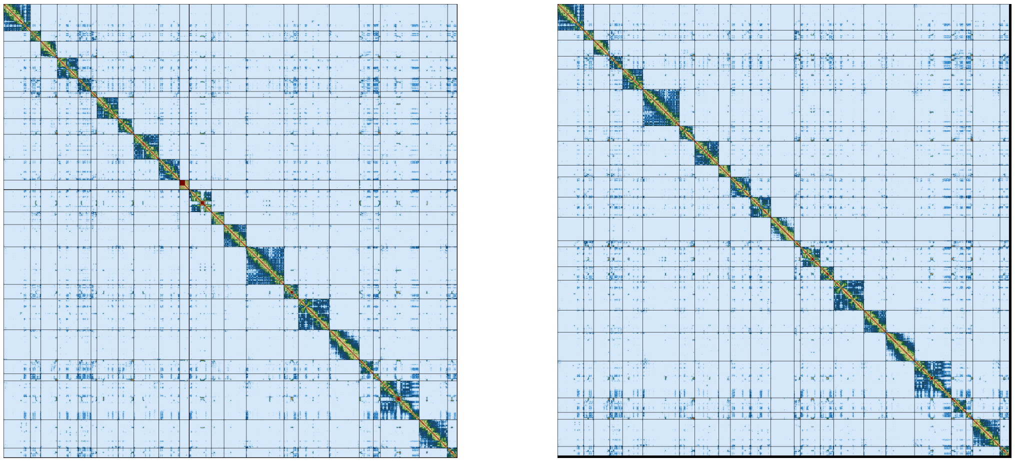

Fig. S4. Hi-C contact maps for HG002 verkko2 hi-c assembly. Curationpretext [3] was used for the map generation. Each haplotype was processed separately.

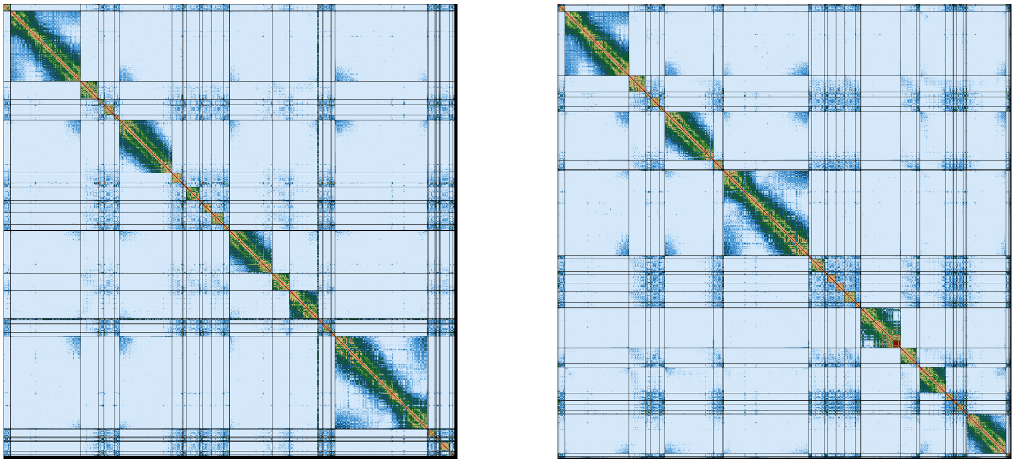

Fig. S5. Hi-C contact maps for chicken verkko2 hi-c assembly. Curationpretext [3] was used for the map generation. Each haplotype was processed separately.

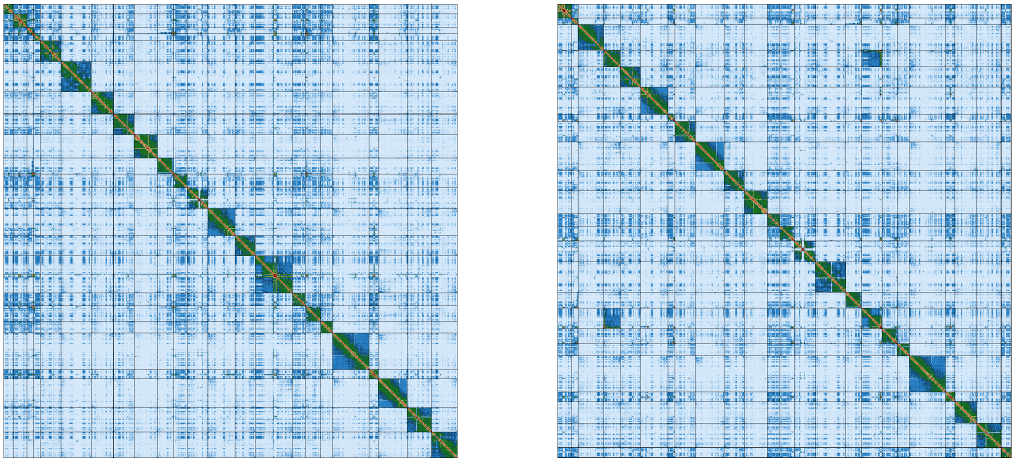

Fig. S6. Hi-C contact maps for HG00733 verkko2 hi-c assembly. Curationpretext [3] was used for the map generation. Each haplotype was processed separately.

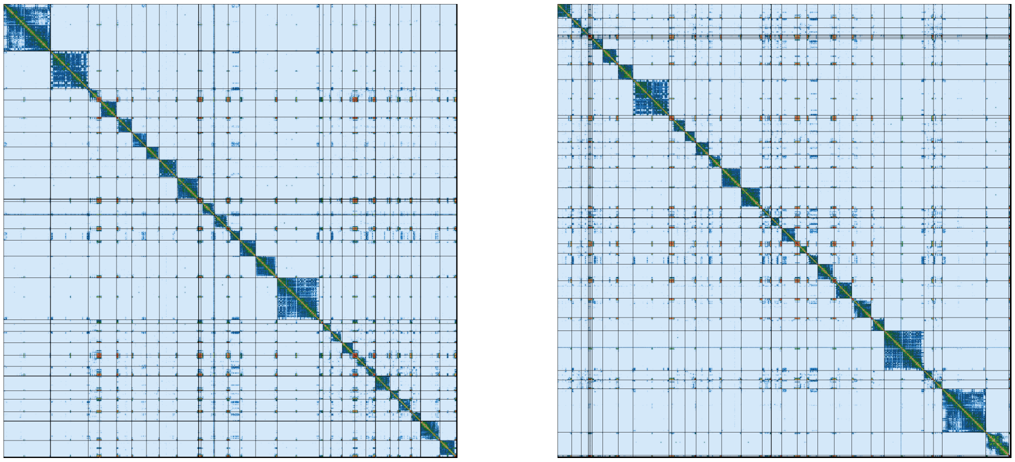

Fig. S7. Hi-C contact maps for sheep verkko2 hi-c assembly. Curationpretext [3] was used for the map generation. Each haplotype was processed separately.

3. SUPPLEMENTARY METHODS

Supplementary methods S1

S1. Overlapper implementation details

The implementation of the overlapper uses a disk index to reduce memory use. To build the index, the overlapper uses several temporary files: one *temporary k-mer file* and multiple *temporary hash files* where the number of temporary hash files f is given as a parameter (default $f = 16$). First all reads are iterated, minimizers are extracted, and their hashes and positions are stored in the temporary k-mer and hash files, respectively. The hashes are divided into the temporary hash files based on their values, with f temporary files placing the hash h into the h mod f'th file. Each temporary hash file is processed one at a time, where the occurrences of each hash value are counted and any hashes appearing only once are discarded. Then, all hashes appearing at least twice are assigned a unique incremental ID, with the first hash in the first file assigned $ID = 0$. The assigned hash IDs are stored in memory as a hash table from k-mer hashes to IDs. Finally, the temporary k-mer file is iterated to build the index file, where the hashes in the temporary k-mer file are replaced with their IDs and the tuple of (ID, read, start, end) is stored in the index file. Due to the read-by-read iteration when reading the k-mers, the k-mers in the index file will have the k-mers of a single read in a contiguous block.

To find the overlaps between the reads, the index file is iterated in multiple batches. Each batch inputs parameters *batch count* c and *batch index* i. The parameters are used to split the reads into c equally large ranges, with the i'th range indexed and matched in one batch. To find the overlaps, the index file is iterated and the k-mers of the reads in the indexed range i are stored in memory. Then, the index file is iterated again and whenever a read in range $x \leq i$ is encountered, all overlaps against the in-memory reads are computed and output. Since the k-mers of each read are in a contiguous block, the k-mers of the matched reads do not need to be stored in memory except for the single read currently being processed, and so the memory use is the index size divided by c. The batch count parameter provides a time-memory trade-off, with more batches requiring less memory but more passes through the index file. In total c batches are required to find all overlaps.

REFERENCES

1. G. W. Vurture, F. J. Sedlazeck, M. Nattestad, *et al.*, "GenomeScope: fast reference-free genome profiling from short reads," Bioinformatics **33**, 2202–2204 (2017).
2. M. Rautiainen, S. Nurk, B. P. Walenz, *et al.*, "Telomere-to-telomere assembly of diploid chromosomes with verkko," Nat. Biotechnol. pp. 1–9 (2023).
3. P. A. Ewels, A. Peltzer, S. Fillinger, *et al.*, "The nf-core framework for community-curated bioinformatics pipelines," Nat. biotechnology **38**, 276–278 (2020).
