# Supplementary material for: Verkko2 integrates proximity-ligation data with long-read De Bruijn graphs for efficient telomere-to-telomere genome assembly, phasing, and scaffolding
Source: Genome Res. 2025 Jul;35(7):1583–94. doi: 10.1101/gr.280383.124 (PMC12212074; doi:10.1101/gr.280383.124)
Supplement: Supplement 1 [file Supplemental_Code.tar.gz › verkko-v2.2.1/src/MBG/parallel-hashmap/benchmark/charts-template.html]

Hash Table Benchmarks


\_\_PLOT\_DIV\_SPEC\_GOES\_HERE\_\_
