# Supplementary material for: Verkko2 integrates proximity-ligation data with long-read De Bruijn graphs for efficient telomere-to-telomere genome assembly, phasing, and scaffolding
Source: Genome Res. 2025 Jul;35(7):1583–94. doi: 10.1101/gr.280383.124 (PMC12212074; doi:10.1101/gr.280383.124)
Supplement: Supplement 1 [file Supplemental_Code.tar.gz › verkko-v2.2.1/src/MBG/parallel-hashmap/html/bench_results/martinus_mod/index2.html]

## Benchmark Results

insert 100m values in map

Lookup 100m ints, all present | Lookup 100m ints, few present
