# Supplementary material for: Verkko2 integrates proximity-ligation data with long-read De Bruijn graphs for efficient telomere-to-telomere genome assembly, phasing, and scaffolding
Source: Genome Res. 2025 Jul;35(7):1583–94. doi: 10.1101/gr.280383.124 (PMC12212074; doi:10.1101/gr.280383.124)
Supplement: Supplement 1 [file Supplemental_Code.tar.gz › verkko-v2.2.1/src/MBG/parallel-hashmap/html/parallel_hashmap.html]

The Parallel Hashmap (Gregory Popovitch)


\[\newcommand{\andalso}{\quad\quad}
\newcommand{\infabbrev}[2]{\infax{#1 \quad\eqdef\quad #2}}
\newcommand{\infrule}[2]{\displaystyle \dfrac{#1}{#2}}
\newcommand{\ar}{\rightarrow}
\newcommand{\Int}{\mathtt{Int}}
\newcommand{\Bool}{\mathtt{Bool}}
\newcommand{\becomes}{\Downarrow}
\newcommand{\trule}[1]{(\textbf{#1})}
\newcommand{\FV}[1]{\mathtt{fv}(#1)}
\newcommand{\FTV}[1]{\mathtt{ftv}(#1)}
\newcommand{\BV}[1]{\mathtt{bv}(#1)}
\newcommand{\compiles}[1]{\text{C}\llbracket{#1}\rrbracket}
\newcommand{\exec}[1]{\text{E}\llbracket{#1}\rrbracket}
\renewcommand{\t}[1]{\mathtt{#1}}
\newcommand{\ite}[3]{\text{if }#1\text{ then }#2\text{ else }#3}
\]

# The Parallel Hashmap

or Abseiling from the shoulders of giants - © Gregory Popovitch - March 10, 2019

[tl;dr] We present a novel hashmap design, the Parallel Hashmap. Built on top of Abseil's *flat\_hash\_map*, the Parallel Hashmap has lower space requirements, is nearly as fast as the underlying *flat\_hash\_map*, and can be used from multiple threads with high levels of concurrency.

### A quick look at the current state of the art

If you haven't been living under a rock, you know that Google open sourced late last year their Abseil library, which includes a very efficient flat hash table implementation. The *absl::flat\_hash\_map* stores the values directly in a memory array, which avoids memory indirections (this is referred to as closed hashing).

Using parallel SSE2 instructions, the flat hash table is able to look up items by checking 16 slots in parallel, which allows the implementation to remain fast even when the table is filled to 87.5% capacity.

The graphs below show a comparison of time and memory usage necessary to insert up to 100 million values (each value is composed of two 8-byte integers), between the default hashmap of Visual Studio 2017 (std::unordered\_map), and Abseil's flat\_hash\_map:

On the bottom graph, we can see that, as expected, the Abseil *flat\_hash\_map* is significantly faster that the default stl implementation, typically about three times faster.

### The peak memory usage issue

The top graph shown the memory usage for both tables.

I used a separate thread to monitor the memory usage, which allows to track the increased memory usage when the table resizes. Indeed, both tables have a peak memory usage that is significantly higher than the memory usage seen between insertions.

In the case of Abseil's *flat\_hash\_map*, the values are stored directly in a memory array. The memory usage is constant until the table needs to resize, which is why we see these horizontal sections of memory usage.

When the *flat\_hash\_map* reaches 87.5% occupancy, a new array of twice the size is allocated, the values are moved (rehashed) from the smaller to the larger array, and then the smaller array, now empty, is freed. So we see that during the resize, the occupancy is only one third of 87.5%, or 29.1%, and when the smaller array is released, occupancy is half of 87.5% or 43.75%.

The default STL implementation is also subject to this higher peak memory usage, since it typically is implemented with an array of buckets, each bucket having a pointer to a linked list of nodes containing the values. In order to maintain O(1) lookups, the array of buckets also needs to be resized as the table size grows, requiring a 3x temporary memory requirement for moving the old bucket array (1x) to the newly allocated, larger (2x) array. In between the bucket array resizes, the default STL implementation memory usage grows at a constant rate as new values are added to the linked lists.

> Instead of having a separate linked list for each bucket, *std::unordered\_map* implementations often use a single linked list (making iteration faster), with buckets pointing to locations within the single linked list. *absl::node\_hash\_map*, on the other hand, has each bucket pointing to a single value, and collisions are handled with open addressing like for the *absl::flat\_hash\_map*.

This peak memory usage can be the limiting factor for large tables. Suppose you are on a machine with 32 GB of ram, and the *flat\_hash\_map* needs to resize when you inserted 10 GB of values in it. 10 GB of values means the array size is 11.42 GB (resizing at 87.5% occupancy), and we need to allocate a new array of double size (22.85 GB), which obviously will not be possible on our 32 GB machine.

For my work developing mechanical engineering software, this has kept me from using flat hash maps, as the high peak memory usage was the limiting factor for the size of FE models which could be loaded on a given machine. So I used other types of maps, such as sparsepp or Google's cpp-btree.

When the Abseil library was open sourced, I started pondering the issue again. Compared to Google's old dense\_hash\_map which resized at 50% capacity, the new *absl::flat\_hash\_map* resizing at 87.5% capacity was more memory friendly, but it still had these significant peaks of memory usage when resizing.

If only there was a way to eliminate those peaks, the *flat\_hash\_map* would be close to perfect. But how?

### The peak memory usage solution

Suddenly, it hit me. I had a solution. I would create a hash table that internally is made of an array of 16 hash tables (the submaps). When inserting or looking up an item, the index of the target submap would be decided by the hash of the value to insert. For example, if for a given `size_t hashval`, the index for the internal submap would be computed with:

`submap_index = (hashval ^ (hashval >> 4)) & 0xF;`

providing an index between 0 and 15.

> In the actual implementation, the size of the array of hash tables is configurable to a power of two, so it can be 2, 4, 8, 16, 32, ... The following illustration shows a parallel\_hash\_map with 8 submaps.

The benefit of this approach would be that the internal tables would each resize on its own when they reach 87.5% capacity, and since each table contains approximately one sixteenth of the values, the memory usage peak would be only one sixteenth of the size we saw for the single *flat\_hash\_map*.

The rest of this article describes my implementation of this concept that I have done inside the Abseil library (I have submitted a pull request in the hope it will be merged into the main Abseil codebase). The current name for it is *parallel\_flat\_hash\_map* or *parallel\_flat\_hash\_set*. It does provide the same external API as Abseil's other hash tables, and internally it uses a std::array of N *flat\_hash\_maps*.

I was delighted to find out that not only the *parallel\_flat\_hash\_map* has significant memory usage benefits compared to the *flat\_hash\_map*, but it also has significant advantages for concurrent programming as I will show later.

> I will use the names *parallel\_hash\_map* and *parallel\_flat\_hash\_map* interchangably. They refer to the same data structure. The name used in my Abseil fork is *absl::parallel\_flat\_hash\_map*, as it may be desirable to also provide a *absl::parallel\_node\_hash\_map*.

### The Parallel Hashmap: memory usage

So, without further ado, let's see the same graphs graphs as above, with the addition of the *parallel\_flat\_hash\_map*. Let us first look at memory usage (the second graph provides a "zoomed-in" view of the location where resizing occurs):

We see that the *parallel\_hash\_map* behaves as expected. The memory usage matches exactly the memory usage of its base *flat\_hash\_map*, except that the peaks of memory usage which occur when the table resizes are drastically reduced, to the point that they are not objectionable anymore. In the "zoomed-in" view, we can see the sixteen dots corresponding to each of the individual submaps resizing. The fact that those resizes are occuring at roughly the same x location in the graph shows that we have a good hash function distribution, distributing the values evenly between the sixteen individual submaps.

### The Parallel Hashmap: speed

But what about the speed? After all, for each value inserted into the parallel hashmap, we have to do some extra work (steps 1 and 2 below):

1. compute the hash for the value to insert
2. compute the index of the target submap from the hash)
3. insert the value into the submap

The first step (compute the hash) is the most problematic one, as it can potentially be costly. As we mentioned above, the second step (computing the index from the hash) is very simple and its cost in minimal (3 processor instruction as shown below in *Matt Godbolt*'s compiler explorer):

As for the hash value computation, fortunately we can eliminate this cost by providing the computed hash to the submap functions, so that it is computed only once. This is exactly what I have done in my implementation of the *parallel\_hash\_map* within the Abseil library, adding a few extra APIs to the Abseil internal raw\_hash\_map.h header, which allow the *parallel\_hash\_map* to pass the precomputed hash value to the underlying submaps.

So we have all but eliminated the cost of the first step, and seen that the cost of the second step is very minimal. At this point we expect that the *parallel\_hash\_map* performance will be close to the one of its underlying *flat\_hash\_map*, and this is confirmed by the chart below:

Indeed, because of the scale is somewhat compressed due to the longer times of the std::unordered\_map, we can barely distinguish between the blue curve of the *flat\_hash\_map* and the red curve of the *parallel\_hash\_map*. So let's look at a graph without the std::unordered\_map:

This last graph shows that the *parallel\_hash\_map* is slightly slower especially for smaller table sizes. For a reason not obvious to me (maybe better memory locality), the speeds of the *parallel\_hash\_map* and *flat\_hash\_map* are essentially undistinguishable for larger map sizes (> 80 million values).

### Are we done yet?

This is already looking pretty good. For large hash\_maps, the *parallel\_flat\_hash\_map* is a very appealing solution, as it provides essentially the excellent performance of the *flat\_hash\_map*, while virtually eliminating the peaks of memory usage which occur when the hash table resizes.

But there is another aspect of the inherent parallelism of the *parallel\_hash\_map* which is interesting to explore. As we know, typical hashmaps cannot be modified from multiple threads without explicit synchronization. And bracketing write accesses to a shared hash\_map with synchronization primitives, such as mutexes, can reduce the concurrency of our program, and even cause deadlocks.

Because the *parallel\_hash\_map* is made of sixteen separate submaps, it posesses some intrinsic parallelism. Indeed, suppose you can make sure that different threads will use different submaps, you would be able to insert into the same *parallel\_hash\_map* at the same time from the different threads without any locking.

### Using the intrinsic parallelism of the *parallel\_hash\_map* to insert values from multiple threads, lock free.

So, if you can iterate over the values you want to insert into the hash table, the idea is that each thread will iterate over all values, and then for each value:

1. compute the hash for that value
2. compute the submap index for that hash
3. if the submap index is one assigned to this thread, then insert the value, otherwise do nothing and continue to the next value

Here is the code for the single-threaded insert:

```
template <class HT>
void _fill_random_inner(int64_t cnt, HT &hash, RSU &rsu)
{
    for (int64_t i=0; i<cnt; ++i)
    {
        hash.insert(typename HT::value_type(rsu.next(), 0));
        ++num_keys[0];
    }
}
```

and here is the code for the multi-threaded insert:

```
template <class HT>
void _fill_random_inner_mt(int64_t cnt, HT &hash, RSU &rsu)
{
    constexpr int64_t num_threads = 8;   // has to be a power of two
    std::unique_ptr<std::thread> threads[num_threads];

    auto thread_fn = [&hash, cnt, num_threads](int64_t thread_idx, RSU rsu) {
        typename HT::hasher hasher;                         // get hasher object from the hash table
        size_t modulo = hash.subcnt() / num_threads;        // subcnt() returns the number of submaps

        for (int64_t i=0; i<cnt; ++i)                       // iterate over all values
        {
            unsigned int key = rsu.next();                  // get next key to insert
            size_t hashval = hash.hash(key);                // compute its hash
            size_t idx  = hash.subidx(hashval);             // compute the submap index for this hash
            if (idx / modulo == thread_idx)                 // if the submap is suitable for this thread
            {
                hash.insert(typename HT::value_type(key, 0)); // insert the value
                ++(num_keys[thread_idx]);                     // increment count of inserted values
            }
        }
    };

    // create and start 8 threads - each will insert in their own submaps
    // thread 0 will insert the keys whose hash direct them to submap0 or submap1
    // thread 1 will insert the keys whose hash direct them to submap2 or submap3
    // --------------------------------------------------------------------------
    for (int64_t i=0; i<num_threads; ++i)
        threads[i].reset(new std::thread(thread_fn, i, rsu));

    // rsu passed by value to threads... we need to increment the reference object
    for (int64_t i=0; i<cnt; ++i)
        rsu.next();
    
    // wait for the threads to finish their work and exit
    for (int64_t i=0; i<num_threads; ++i)
        threads[i]->join();
}
```

Using multiple threads, we are able to populate the *parallel\_flat\_hash\_map* (inserting 100 million values) three times faster than the standard *flat\_hash\_map* (which we could not have populated from multiple threads without explicit locks, which would have prevented performance improvements).

And the graphical visualization of the results:

We notice in this last graph that the memory usage peaks, while still smaller than those of the *flat\_hash\_map*, are larger that those we saw when populating the *parallel\_hash\_map* using a single thread. The obvious reason is that, when using a single thread, only one of the submaps would resize at a time, ensuring that the peak would only be 1/16th of the one for the *flat\_hash\_map* (provided of course that the hash function distributes the values somewhat evenly between the submaps).

When running in multi-threaded mode (in this case eight threads), potentially as many as eight submaps can resize simultaneaously, so for a *parallel\_hash\_map* with sixteen submaps the memory peak size can be half as large as the one for the *flat\_hash\_map*.

Still, this is a pretty good result, we are now inserting values into our *parallel\_hash\_map* three times faster than we were able to do using the *flat\_hash\_map*, while using a lower memory ceiling.

This is significant, as the speed of insertion into a hash map is important in many algorithms, for example removing duplicates in a collection of values.

### Using the intrinsic parallelism of the *parallel\_hash\_map* with internal mutexes

It may not be practical to add logic into your program to ensure you use different internal submaps from each thread. Still, locking the whole *parallel\_hash\_map* for each access would forego taking advantage of its intrinsic parallelism.

For that reason, the *parallel\_hash\_map* can provide internal locking using the `absl::Mutex` (the default template parameter is `absl::NullMutex`, which does no locking and has no size cost). When selecting `absl::Mutex`, one mutex is created for each internal submap at a cost of 8 bytes per submap, and the *parallel\_hash\_map* internally protects each submap access with its associated mutex.

| map | Number of submaps | sizeof(map) |
| --- | --- | --- |
| std::unordered\_map (vs2017) | - | 64 |
| absl::flat\_hash\_map | - | 48 |
| absl::parallel\_flat\_hash\_map, N=4, absl::NullMutex | 16 | 768 |
| absl::parallel\_flat\_hash\_map, N=4, absl::Mutex | 16 | 896 |

It is about time we provide the complete parallel\_flat\_hash\_map class declaration (the declaration for parallel\_flat\_hash\_set is similar):

```
template <class K, class V,
          class Hash      = absl::container_internal::hash_default_hash<K>,
          class Eq        = absl::container_internal::hash_default_eq<K>,
          class Allocator = std::allocator<std::pair<const K, V>>,
          size_t N        = 4,                 // 2**N submaps
          class Mutex     = absl::NullMutex>   // use absl::Mutex to enable internal locks
class parallel_flat_hash_map;
```

Let's see what result we get for the insertion of random values from multiple threads, however this time we create a *parallel\_hash\_map* with internal locking (by providing absl::Mutex as the last template argument), and modify the code so that each thread inserts values in any submap (no pre-selection).

If we were to do a intensive insertion test into a hash map from multiple threads, where we lock the whole hash table for each insertion, we would be likely to get even worse results than for a single threaded insert, because of heavy lock contention.

In this case, our expectation is that the finer grained locking of the *parallel\_hash\_map* (separate locks for each internal submap) will provide a speed benefit when compared to the single threaded insertion, and this is indeed what the benchmarks show:

Interestingly, we notice that the memory peaks (when resizing occur) are again very small, in the order of 1/16th of those for the *flat\_hash\_map*. This is likely because, as soon as one of the submaps resizes (which takes much longer than a regular insertion), the other threads very soon have to wait on the resizing submap's mutex for an insertion, before they reach their own resizing threashold.

Since threads statistically will insert on a different submap for each value, it would be a surprising coincidence indeed if two submaps reached their resizing threshold without the resizing of the first submap blocking all the other threads first.

If we increase the number of submaps, we should see more parallelism (less lock contention across threads, as the odds of two separate threads inserting in the same subhash is lower), but with diminishing returns as every submap resize will quickly block the other threads until the resize is completed.

This is indeed what we see:

| map | Number of submaps | sizeof(map) | time 100M insertions |
| --- | --- | --- | --- |
| absl::flat\_hash\_map | - | 48 | 14.77s |
| absl::parallel\_flat\_hash\_map, N=4, absl::Mutex | 16 | 896 | 8.36s |
| absl::parallel\_flat\_hash\_map, N=5, absl::Mutex | 32 | 1792 | 7.14s |
| absl::parallel\_flat\_hash\_map, N=6, absl::Mutex | 64 | 3584 | 6.61s |

There is still some overhead from the mutex lock/unlock, and the occasional lock contention, which prevents us from reaching the performance of the previous multithreaded lock-free insertion (5.12s for inserting 100M elements).

### In Conclusion

We have seen that the novel parallel hashmap approach, used within a single thread, provides significant space advantages, with a very minimal time penalty. When used in a multi-thread context, the parallel hashmap still provides a significant space benefit, in addition to a consequential time benefit by reducing (or even eliminating) lock contention when accessing the parallel hashmap.

### Future work

1. It would be beneficial to provide additional APIs for the *parallel\_flat\_hash\_map* and *parallel\_flat\_hash\_set* taking a precomputed hash value. This would enable the lock-free usage of the *parallel\_flat\_hash\_map*, described above for multi-threaded environments, without requiring a double hash computation.
2. We may consider providing *parallel\_node\_hash\_map* and *parallel\_node\_hash\_set* in Abseil, for the cases when pointer stability is required for keys and/or values. This would be a simple addition.

### Thanks

I would like to thank Google's *Matt Kulukundis* for his eye-opening presentation of the *flat\_hash\_map* design at CPPCON 2017 - my frustration with not being able to use it helped trigger my insight into the *parallel\_hash\_map*. Also many thanks to the Abseil container developers - I believe the main contributors are *Alkis Evlogimenos* and *Roman Perepelitsa* - who created an excellent codebase into which the graft of this new hashmap took easily, and finally to Google for open-sourcing Abseil. Thanks also to my son *Andre* for reviewing this paper, and for his patience when I was rambling about the *parallel\_hash\_map* and its benefits.

### Links

Github repository for the benchmark code used in this paper

Swiss Tables doc

My fork of Google Abseil repository, with the parallel\_flat\_hash\_map implementation

Google Abseil repository

Matt Kulukindis: Designing a Fast, Efficient, Cache-friendly Hash Table, Step by Step
