# Supplementary material for: Verkko2 integrates proximity-ligation data with long-read De Bruijn graphs for efficient telomere-to-telomere genome assembly, phasing, and scaffolding
Source: Genome Res. 2025 Jul;35(7):1583–94. doi: 10.1101/gr.280383.124 (PMC12212074; doi:10.1101/gr.280383.124)
Supplement: Supplement 1 [file Supplemental_Code.tar.gz › verkko-v2.2.1/src/MBG/parallel-hashmap/html/parallel_hashmap.pdf]

Contents

the parallel hashmap 2

% The Parallel Hashmap % Gregory Popovitch % March 3, 2019

the parallel hashmap

(or Abseiling from the shoulders of giants)

(c) Gregory Popovitch - 2/28/2019

[tl;dr] built on top of Abseil's flat_hash_map, the parallel flat_hash_map is more memory friendly, and can be used from multiple threads with high levels of concurrency

A quick look at the current state of the art

If you haven't been living under a rock, you know that Google open sourced late last year their Abseil library, which includes a very efficient flat hash table implementation. The absl::flat_hash_map stores the values directly in a memory array, which avoids memory indirections (this is referred to as closed hashing).

closed_hashing

Using parallel SSE2 instructions, the flat hash table is able to look up items by checking 16 slots in parallel, which allows the implementation to remain fast even when the table is filled to 87.5% capacity.

The graphs below show a comparison of time and memory usage necessary to insert up to 100 million values (each value is composed of two 8-byte integers), between the default hashmap of Visual Studio 2017 (std::unordered_map), and Abseil's flat_hast_map:

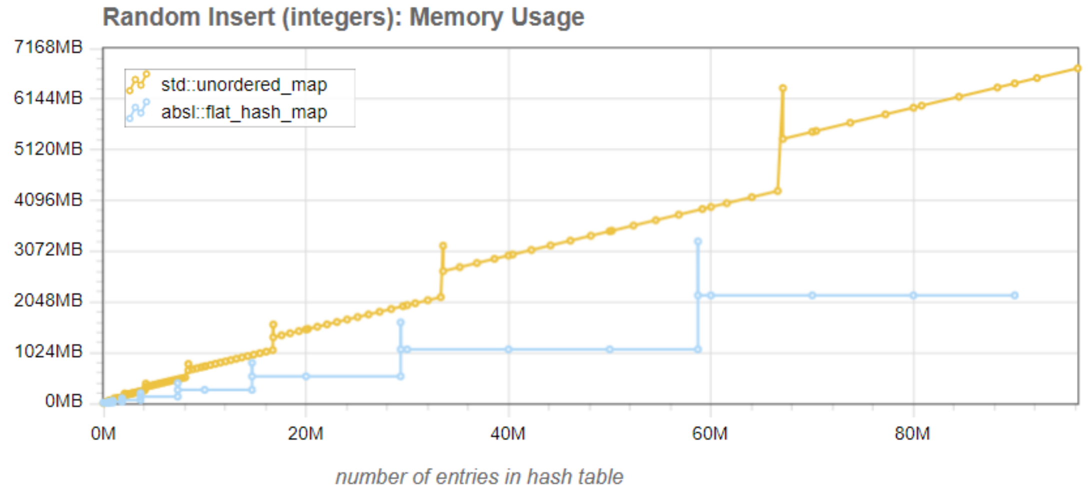

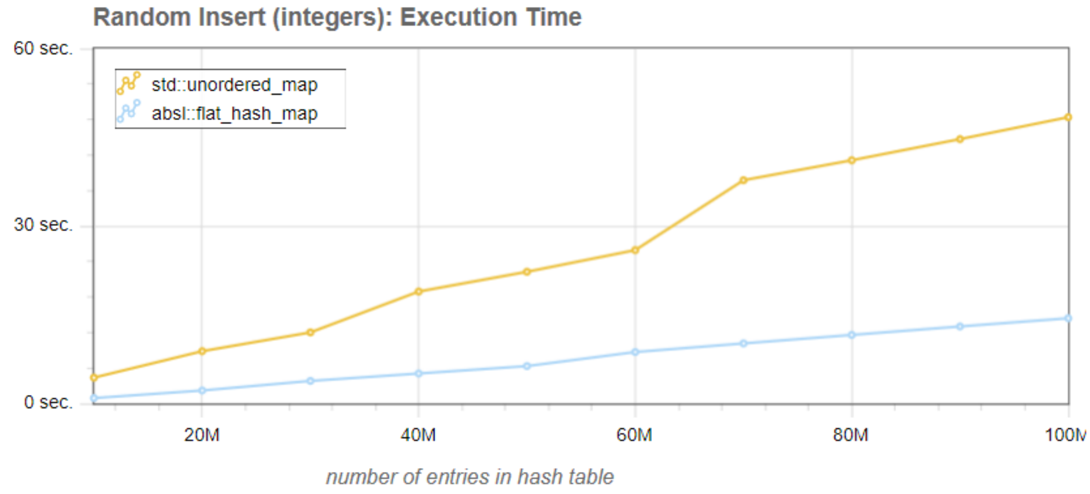

On the bottom graph, we can see that, as expected, the Abseil flat_hash_map is significantly faster that the default stl implementation, typycally about three times faster.

Peak memory usage: the issue

The top graph shown the memory usage for both tables.

I used a separate thread to monitor the memory usage, which allows to track the increased memory usage when the table resizes. Indeed, both tables have a peak memory usage that is significantly higher than the memory usage seen between insertions.

In the case of Abseil's flat_hash_map, the values are stored directly in a memory array. The memory usage is constant until the table needs to resize, which is what we see with these horizontal sections of memory usage.

When the flat_hash_map reaches 87.5% occupancy, a new array of twice the size is allocated, the values are moved (rehashed) from the smaller to the larger array, and then the smaller array, now empty, is freed. So we see that during the resize, the occupancy is only one third of 87.5%, or 29.1%, and when the smaller array is released, occupancy is half of 87.5% or 43.75%.

The default STL implementation is also subject to this higher peak memory usage, since it typically is implemented with an array of buckets, each bucket having a pointers to a linked list of nodes containing the values. In order to maintain O(1) lookups, the array of buckets also needs to be resized as the table size grows, requiring a 3x temporary memory requirement for moving the old bucket array (1x) to the newly allocated, larger (2x) array. In between the bucket array resizes, the default STL implementation memory usage grows at a constant rate as new values are added to the linked lists.

This peak memory usage can be the limiting factor for large tables. Suppose you are on a machine with 32 GB of ram, and the flat_hash_map needs to resize when you inserted 10 GB of values in it. 10 GB of values means the array size is 11.42 GB (resizing at 87.5% occupancy), and we need to allocate a new array of double size (22.85 GB), which obviously will not be possible on our 32 GB machine.

For my work developing mechanical engineering software, this has kept me from using flat hash maps, as the high peak memory usage was the limiting factor for the size of FE models which could be loaded on a given machine. So I used other types of maps, such as sparsepp or Google's cpp-btree.

When the Abseil library was open sourced, I started pondering the issue again. Compared to Google's old dense_hash_map which resized at 50% capacity, the new absl::flat_hash_map resizing at 87.5% capacity was more memory friendly, but it still had those dreadful peaks of memory usage when resizing.

If only there was a way to eliminate those peaks, the flat_hash_map would be close to perfect. But how?

Peak memory usage: the solution

Suddenly, it hit me. I had a solution. I would create a hash table that internally is made of an array of 16 hash tables (the submaps). When inserting or looking up an item, the index of the target submap would be decided by the hash of the value to insert. For example, if for a given `size_t hashval`, the index for the inner submap would be computed with:

```
submap_index = (hashval ^ (hashval >> 4)) & 0xF;
```

providing an index between 0 and 15.

> In the actual implementation, the size of the array of hash tables is configurable to a power of two, so it can be 2, 4, 8, 16, 32, ... The following illustration shows a parallel_hash_map with 8 submaps.

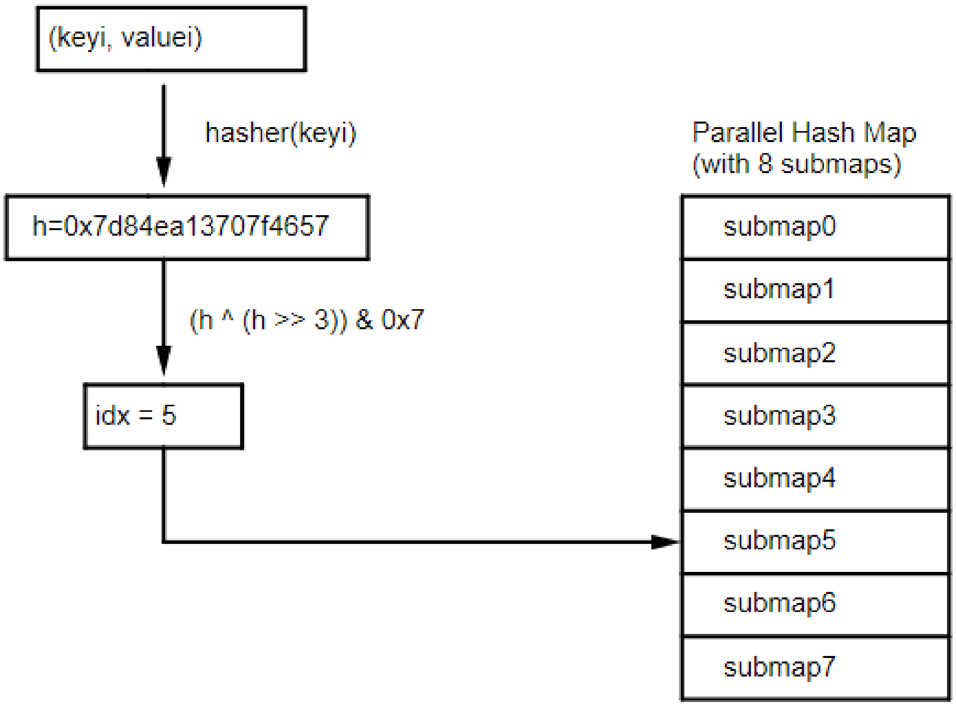

parallel_hash_map with 8 submaps, each submap is an absl::flat_hash_map

The benefit of this approach would be that the internal tables would each resize on its own when they reach 87.5% capacity, and since each table contains approximately one sixteenth of the values, the memory usage peak would be only one sixteenth of the size we saw for the single flat_hash_map.

The rest of this article describes my implementation of this concept that I have done inside the Abseil library (I have submitted a pull request in the hope it will be merged into the main Abseil codebase). THe current name for it is `parallel_flat_hash_map` or `parallel_flat_hash_set`. It does provide the same external API as Abseils other hash tables, and internally it uses a std::array of N flat_hash_maps.

I was delighted to find out that not only the parallel_flat_hash_map has significant memory usage benefits compared to the flat_hash_map, but it also has significant advantages for concurrent programming as I will show later.

> I will use the names parallel_hash_map and parallel_flat_hash_map interchangably. They refer to the same data structure. The name used in my Abseil fork is absl::parallel_flat_hash_map, as it may be desirable to also provide a absl::parallel_node_hash_map.

The parallel_hash_map: memory usage

So, without further ado, let's see the same graphs graphs as above, with the addition of the parallel_flat_hash_map. Let us first look at memory usage (the second graph provides a "zoomed-in" view of the location where resizing occurs):

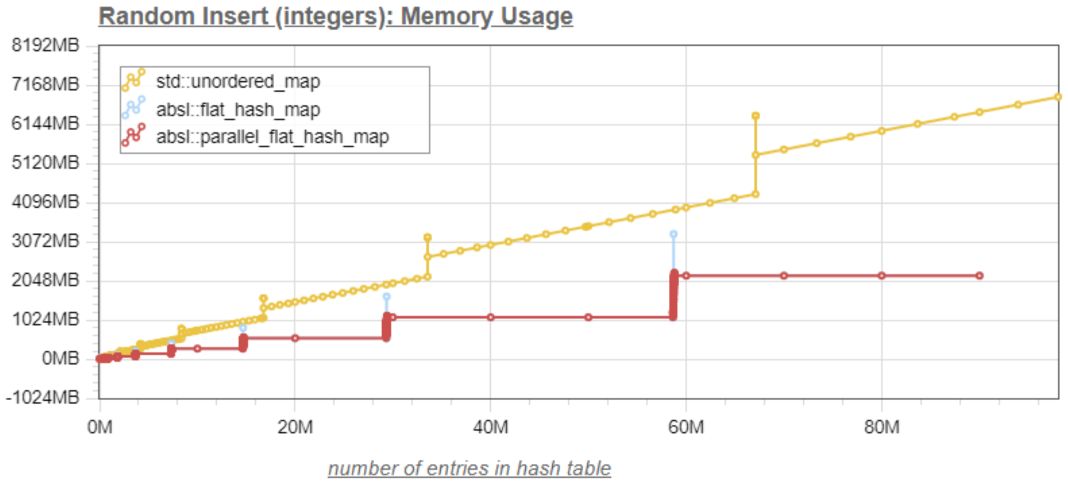

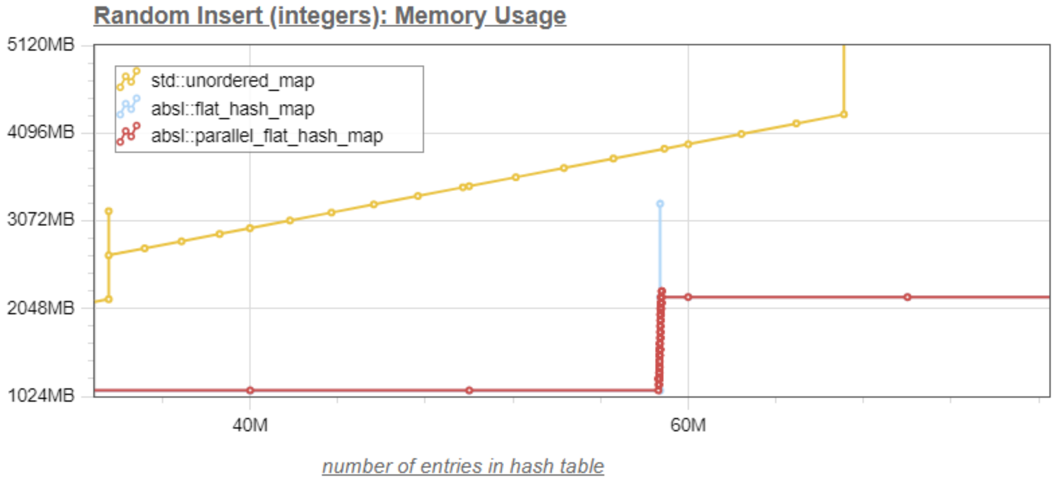

We see that the parallel_hash_map behaves as expected. The memory usage matches exactly the memory usage of its base flat_hash_map, except that the peaks of memory usage which occur when the table resizes are drastically reduced, to the point that they are not objectionable anymore. In the "zoomed-in" view, we can see the sixteen dots corresponding to each of the individual sub-tables resizing. The fact that those resizes are occuring at roughly the same x location in the graph shows that we have a good

hash function distribution, distributing the values evenly between the sixteen individual submaps.

The parallel_hash_map: speed

But what about the speed? After all, for each value inserted into the parallel hashmap, we have to do some extra work (steps 1 and 2 below):

1. compute the hash for the value to insert
2. compute the index of the target sub-table from the hash)
3. insert the value into the sub-table

The first step (compute the hash) is the most problematic one, as it can potentially be costly. As we mentioned above, the second step (computing the index from the hash) is very simple and its cost in minimal (3 processor instruction as shown below in Matt Godbolt's compiler explorer):

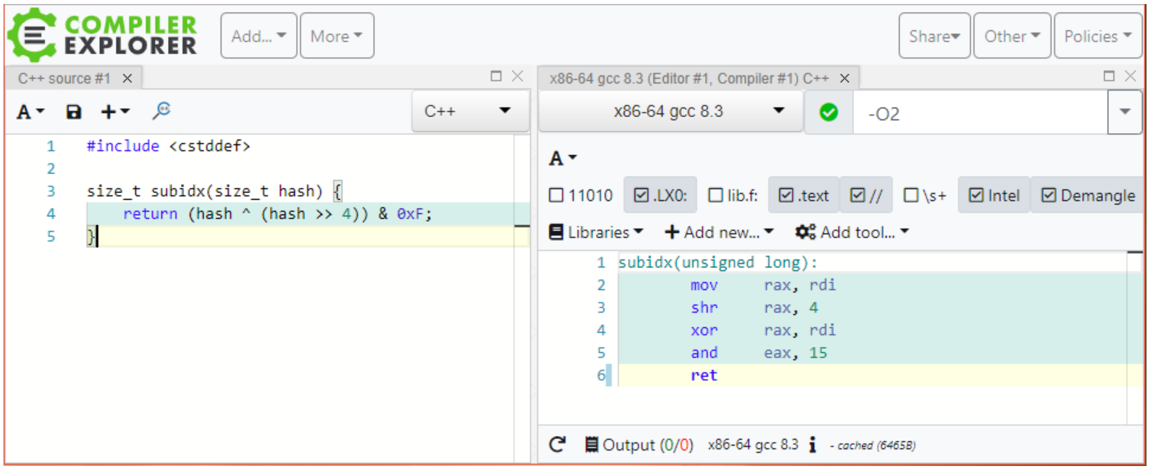

As for the hash value computation, fortunately we can eliminate this cost by providing the computed hash to the sub-table functions, so that it is computed only once. This is exactly what I have done in my implementation pof the parallel_hash_map withing the Abseil library, adding a few extra APIs to the Abseil internal raw_hash_map.h header,= which allow the parallel_hash_map to pass the precomputed hash value to the underlying hash tables.

So we have all but eliminated the cost of the first step, and seen that the cost of the second step is very minimal. At this point we expect that the parallel_hash_map performance will be close to the one of its underlying flat_hash_map, and this is confirmed by the chart below:

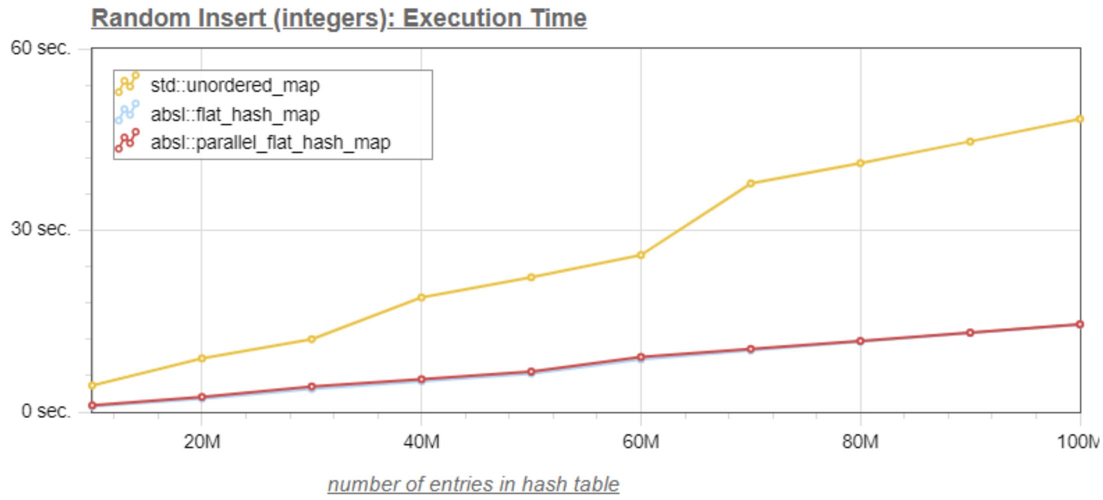

Indeed, because of the scale is somewhat compressed due to the longer times of the std::unordered_map, we can barely distinguish between the blue curve of the flat_hash_map and the red curve of the parallel_hash_map. So let's look at a graph without the std::unordered_map:

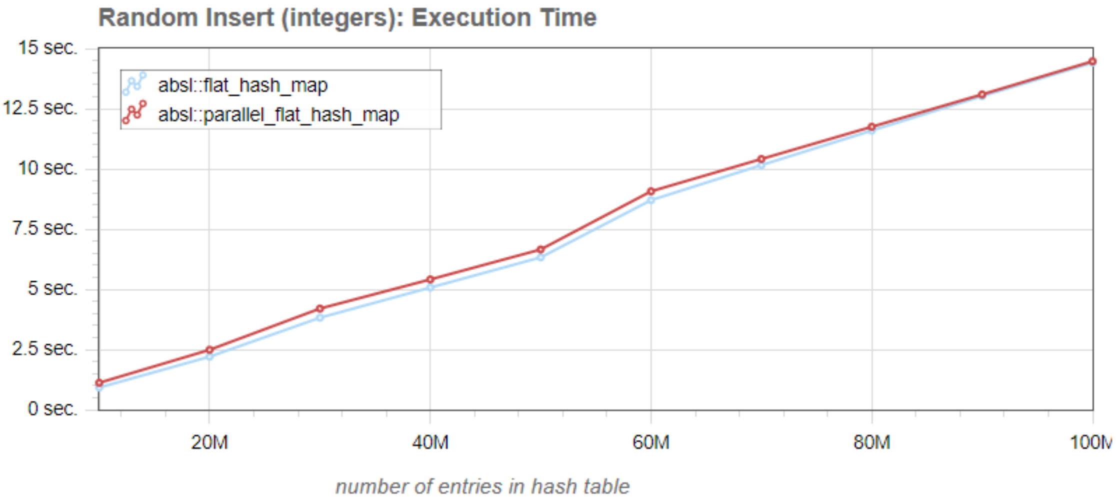

This last graph that the parallel_hash_map is slightly slower especially for smaller table sizes. For a reason not obvious to me (maybe better memory locality), the speeds of the parallel_hash_map and flat_hash_map are essentially undistinguishable for larger map sizes (> 80 million values).

Are we done yet?

This is already looking pretty good. For large hash_maps, the parallel_flat_hash_map is a very appealing solution, as it provides essentially the excellent performance of the flat_hash_map, while virtually eliminating the peaks of memory usage which occur when the hash table resizes.

But there is another aspect of the inherent parallelism of the parallel_hash_map which is interesting to explore. As we know, typical hashmaps cannot be modified from multiple threads without explicit synchronization. And bracketing write accesses to a shared hash_map with synchronization primitives, such as mutexes, can reduce the concurrency of our program, and even cause deadlocks.

Because the parallel_hash_map is built of sixteen separate submaps, it posesses some intrinsic parallelism. Indeed, suppose you can make sure that different threads will use different submaps, you would be able to insert into the same parallel_hash_map at the same time from the different threads without any locking.

Using the intrinsic parallelism of the parallel_hash_map to insert values from multiple threads, lock free.

So, if you can iterate over the values you want to insert into the hash table, the idea is that each thread will iterate over all values, and then for each value:

1. compute the hash for that value
2. compute the submap index for that hash
3. if the submap index is one assigned to this thread, then insert the value, otherwise do nothing and continue to the next value

Here is the code for the single-threaded insert:

```
template <class HT>
void _fill_random_inner(int64_t cnt, HT &hash, RSU &rsu)
{
    for (int64_t i=0; i<cnt; ++i)
    {
        hash.insert(typename HT::value_type(rsu.next(), 0));
        ++num_keys[0];
    }
}
```

and here is the code for the multi-threaded insert:

```
template <class HT>
void _fill_random_inner_mt(int64_t cnt, HT &hash, RSU &rsu)
{
    constexpr int64_t num_threads = 8;   // has to be a power of two
```

```
    std::unique_ptr<std::thread> threads[num_threads];

    auto thread_fn = [&hash, cnt, num_threads](int64_t thread_idx, RSU rsu) {
        typename HT::hasher hasher;                         // get hasher object from the hash ta
        size_t modulo = hash.subcnt() / num_threads;        // subcnt() returns the number of sub

        for (int64_t i=0; i<cnt; ++i)                       // iterate over all values
        {
            unsigned int key = rsu.next();                  // get next key to insert
            size_t hashval = hasher(key);                   // compute its hash
            size_t idx  = hash.subidx(hashval);             // compute the submap index for this
            if (idx / modulo == thread_idx)                 // if the submap is suitable for this
            {
                hash.insert(typename HT::value_type(key, 0)); // insert the value
                ++(num_keys[thread_idx]);                     // increment count of inserted valu
            }
        }
    };

    // create and start 8 threads - each will insert in their own submaps
    // thread 0 will insert the keys whose hash direct them to submap0 or submap1
    // thread 1 will insert the keys whose hash direct them to submap2 or submap3
    // --------------------------------------------------------------------------
    for (int64_t i=0; i<num_threads; ++i)
        threads[i].reset(new std::thread(thread_fn, i, rsu));

    // rsu passed by value to threads... we need to increment the reference object
    for (int64_t i=0; i<cnt; ++i)
        rsu.next();

    // wait for the threads to finish their work and exit
    for (int64_t i=0; i<num_threads; ++i)
        threads[i]->join();
}
```

Using multiple threads, we are able to populate the parallel_flat_hash_map (inserting 100 million values) three times faster than the standard flat_hash_map (which we could not have populated from multiple threads without explicit locks, which would have prevented performance improvements).

And the graphical visualization of the results:

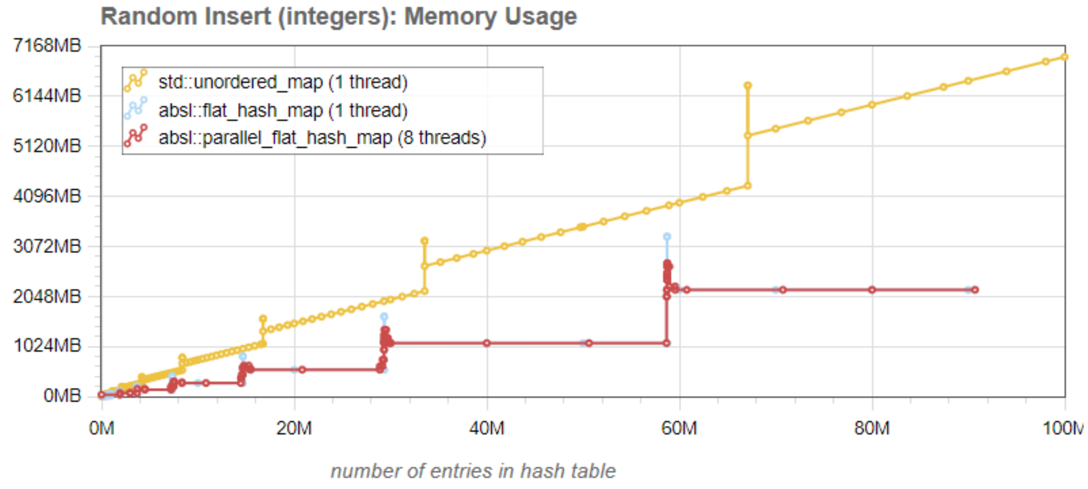

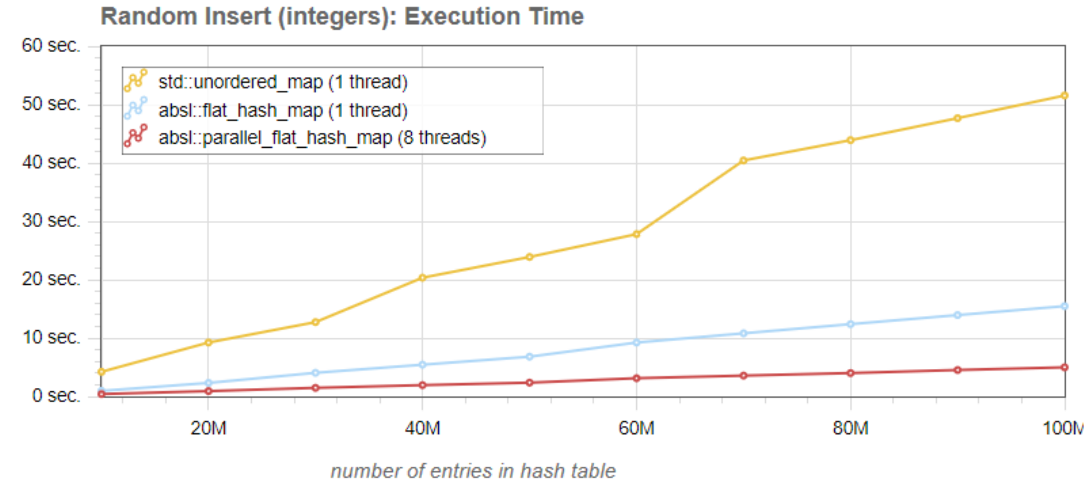

We notice in this last graph that the memory usage peaks, while still smaller than those of the flat_hast_map, are larger that those we saw when populating the parallel_hash_map using a single thread. The obvious reason is that, when using a single thread, only one of the submaps would resize at a time, ensuring that the peak would only be 1/16th of the one for the flat_hash_map (provided of course that the hash function distributes the values somewhat evenly between the submaps).

When running in multi-threaded mode (in this case eight threads), potentially as many as eight submaps can resize simultaneaously, so for a parallel_hash_map with sixteen submaps the memory peak size can be half as large as the one for the flat_hash_map.

Still, this is a pretty good result, we are now inserting values into our parallel_hash_map three times faster than we were able to do using the flat_hash_map, while using a lower memory ceiling.

In Conclusion

We have seen that the novel parallel hashmap approach, used withing a single thread, provides significant space advantages, with a very minimal time penalty. When used in a multi-thread context, the parallel hashmap still provides a significant space benefit, in addition to a time benefit by drastically reducing (or even eliminating) lock contention when accessing the parallel hashmap.

Thanks

I would like to thank Google's Matt Kulukundis for his excellent presentation of the flat_hash_map design at CPPCON 2017 - my frustration with not being able to use it helped trigger my insight into the parallel_map_map. Also many thanks to the Abseil container developers - I believe the main contributors are Alkis Evlogimenos and Roman Perepelitsa - who created an excellent codebase into which the graft of this new hashmap took easily, and finally to Google for open-sourcing Abseil. Thanks also to my son Andre for reviewing this paper, and for his patience when I was rambling to him about the parallel_hash_map and all its benefits.

Links

github repository for the benchmark code used in this paper

Swiss Tables doc

Google Abseil repository

Matt Kulukindis: Designing a Fast, Efficient, Cache-friendly Hash Table, Step by Step
