# Supplementary material for: Verkko2 integrates proximity-ligation data with long-read De Bruijn graphs for efficient telomere-to-telomere genome assembly, phasing, and scaffolding
Source: Genome Res. 2025 Jul;35(7):1583–94. doi: 10.1101/gr.280383.124 (PMC12212074; doi:10.1101/gr.280383.124)
Supplement: Supplement 1 [file Supplemental_Code.tar.gz › verkko-v2.2.1/src/MBG/parallel-hashmap/html/template.html]

The Parallel Hashmap (Gregory Popovitch)


$if(highlighting-css)$
$endif$
$for(css)$
$endfor$
$if(math)$
$if(html5)$
$else$
$math$
$endif$
$endif$
$for(header-includes)$
$header-includes$
$endfor$


$body$
